# Studying cancer immunotherapy using patient-derived xenografts (PDXs) in humanized mice

**DOI:** 10.1038/s12276-018-0115-0

**Published:** 2018-08-07

**Authors:** Yunsik Choi, Sanghyuk Lee, Kapyoul Kim, Soo-Hyun Kim, Yeun-Jun Chung, Charles Lee

**Affiliations:** 1 Department of Life Sciences, Ewha Womans University, Seoul, Korea; 2 Department of Microbiology, Precision Medicine Research Center, IRCGP, College of Medicine, The Catholic University of Korea, Seoul, Korea; 3 The Jackson Laboratory for Genomic Medicine, Farmington, CT USA

## Abstract

Cancer immunotherapy is a promising way to eliminate tumor cells by using the patient’s own immune system. Selecting the appropriate animal models to develop or validate preclinical immunotherapeutic trials is now an important aspect of many cancer research programs. Here we discuss the advantages and limitations of using genetically engineered immunodeficient mouse models, patient-derived xenografts (PDXs), and humanized mouse models for developing and testing immunotherapeutic strategies.

## Cancer immunotherapy: Improving animal models

Improvements to mouse models for cancer immunotherapy could enhance the precision of new drugs. Immunotherapy trials require genetically modified animal models, including ‘humanized’ mice with a functioning human immune system, and patient-derived xenograft (PDX) mice, in which cells from patients’ tumors are implanted into immunodeficient mice. Charles Lee at the Jackson Laboratory for Genomic Medicine in Farmington, USA, Yeun-Jun Chung at the Catholic University of Korea in Seoul, and co-workers reviewed developments in both PDX and humanized-PDX mouse models for immunotherapy trials. PDX models improve the chances of finding novel biomarkers for drug development. However, humanized PDX mouse models will allow researchers to study diverse cancers in tumour and immune environments as close as possible to those of humans.

## Introduction

Immune surveillance against cancer is an important protection of the host to restrain carcinogenesis and sustain cellular homeostasis. Immune surveillance has three essential phases: elimination, equilibrium, and escape^1^. During the interaction of host cells with tumor cells, the immune system is usually capable of recognizing tumor cells from novel cell surface antigens that arise from genetic and/or epigenetic changes in the tumor cells. To overcome this immune surveillance, tumor cells can use a variety of mediators for immunosuppression. For example, T-cell activation can be suppressed by adenosine and vascular endothelial growth factor A, which are released by tumor cells under hypoxia. Another mechanism for immune suppression is when tumor cells downregulate major histocompatibility complex (MHC) class I (reducing the presentation of intracellular peptide fragments on the cell surfaces of tumor cells) or disable other components of the antigen-presenting process, to escape T-cell recognition^2, 3^.

The concept of cancer immunotherapy was initiated by William B. Coley, who observed tumor shrinkage and disappearance following treatment with a bacterial toxin in the 1890s. Since then, immunotherapy has subsequently developed into a novel method for treating cancer by reinforcing the immune system, rather than attacking the tumor cells directly with chemotherapeutic agents. Immunotherapy treatment can be broadly classified as either cancer vaccines, adoptive cellular immunotherapy, or therapies using immune checkpoint blockades^4^.

To perform systematic preclinical cancer immunotherapy studies, it is important to select appropriate animal models. Various animal models, namely, genetically engineered mice, patient-derived xenografts (PDXs), and humanized PDX mouse models, can be used for testing new anticancer immunotherapies. In this review, we discuss the basic principle of PDXs and humanized mouse models and their applications in cancer research. We also discuss the limitations of these models and review strategies that may be used to overcome these limitations.

## Cancer immunotherapy

The host immune system has an important role in tumor development and control. Cancer immunotherapy uses the immune system to eliminate tumor cells. Currently, there are various approaches to cancer immunotherapy, including cancer vaccines, adoptive cell therapies (ACT) and immune checkpoint blockade therapies (Table 1).

**Table 1:** Categories of cancer immunotherapies

Strategy	Basic mechanism and major advantages	Major disadvantages	References
Vaccines	Enhances CD4^+^ and CD8^+^ T-cell responses Some success has been reported, such as for cervical cancer.	Free peptides are rapidly cleared from the body before binding to antigen-presenting cells (APCs). Most antigens in cancer cells are also found in normal cells (i.e., not cancer-cell specific). There can be insufficient immunization and responses to a specific tumor antigen.	^3, 5, 6^
Adoptive cell therapy (ACT)	Tumor infiltrated lymphocytes (TILs) trigger tumor cell death and eradicate the tumor. Engineered T-cell receptors (TCRs), with a high affinity and specificity for tumor antigens, are capable of activating T cells to target cancer cells. Adoptive transfer of lymphocytes generates a high avidity in effector T cells.	Not all patients can participate in these clinical trials, as they are generally restricted to patients capable of undergoing lymphodepletion- and IL-2-based treatments. Mispairing the engineered TCR α- and β-chains can occur in the engineered T cell. There have been safety issues encountered, manufacturing is complex and costs remain high.	^3, 15, 16^
Immune checkpoint blockade	This method enhances preexisting anticancer immune responses that are dependent on T cells. There is the potential for a long-term survival following treatment. It has been shown to be applicable to multiple cancer types.	T-cell responses could take several months to occur There is a significant amount of toxicity observed among patients. A minority of patients, treated with immune checkpoint blockade therapy, actually experience a durable response.	^3, 25, 26^

### Cancer vaccines

Cancer vaccines can be classified into the following two categories: prophylactic and therapeutic^5^. Prophylactic vaccines have been used with considerable success for the prevention of cancers of viral origin such as for cervical and liver cancers^6, 7^. Therapeutic vaccines have been developed to specifically stimulate CD8^+^ and CD4^+^ T cells. These vaccines can then target the differentiated antigens expressed on the cell surfaces of tumor cells^8^. However, cancer vaccines, consisting of short peptides, can be rapidly cleared before being loaded onto antigen-presenting cells (APCs). In other cases, the therapeutic benefits can be limited by an insufficient immunization or response to a selected tumor antigen^3^. Finally, these vaccines require knowledge and purification of tumor-specific antigens. Since this is still quite limited, this technology is currently applicable to only a few types of cancers and stages^9^.

Dendritic cells (DCs) have been shown to be more efficient at antigen presentation and the induction of T-cell immunity compared to other APCs such as macrophages. In this approach, DCs are isolated from the patient’s peripheral blood mononuclear cells (PBMC), loaded with tumor antigens ex vivo, activated, and then reinfused back into the patient^10, 11^. Indeed, DC vaccinations have already produced some meaningful clinical results in a subset of patients with advanced cancers^12^. For instance, treatment with sipuleucel-T (a cellular product based on enriched blood APCs briefly cultured with a prostatic acid phosphatase fusion protein) achieved an approximate four-month improvement in the median survival for some patients with metastatic prostate cancer^13, 14^.

### Adoptive cell therapy

The treatment of cancer patients with immune cells isolated from the body, expanded ex vivo, and reinjected into the patient for tumor cell targeting that ultimately induces cell death is called ACT. In the host, T-cell-mediated antitumor immune responses can be triggered by APCs that capture antigens from tumor cells. T cells then scan for unrecognized MHC-peptide complexes, which would alert them to potentially threatening foreign antigens and then lead to the activation of their T-cell receptors^15^. Tumor cells generally express antigens that are characteristic of their tissues of origin, and tissue-differentiated antigens are attractive targets for ACT.

For immunotherapies based on the adoptive transfer of tumor infiltrated lymphocytes (TILs), genetically engineered T cells that express T-cell receptors (TCRs) with a high affinity and specificity for target antigens would be an excellent treatment option^16–20^. Chimeric antigen receptors (CARs) are one way for providing specificity to transduced T cells (CAR-T cells) and can originate from antibodies. CARs recognize MHC-nonrestricted structures on the surfaces of target cells, whereas TCRs recognize mainly intracellular antigens that have been presented as peptide complexes with MHC molecules^21, 22^. The genetic modification of T cells for ACT can confer new antigenicity to recipient T cells. However, there are several limitations to this method. For example, current approaches have only monoclonal specificity and may only be effective for the treatment of a small proportion of the tumor cells. In the case of genetically engineered TCRs, mispairing of TCRs with endogenous TCR chains can also occur^23^.

### Immune checkpoint blockade therapy

**Fig. 1:**
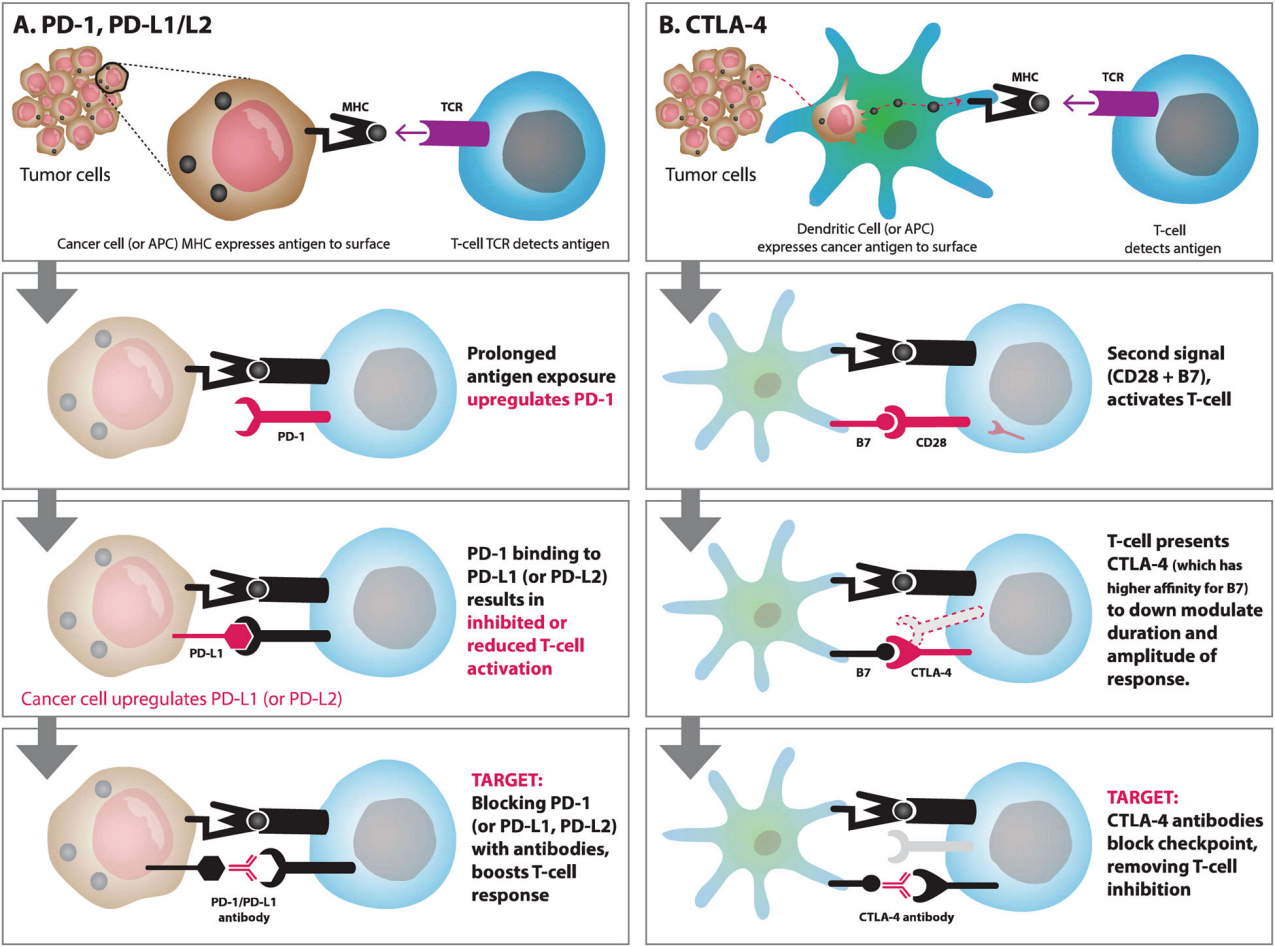
Regulation of antitumor immunity and immunotherapy targets. **a** Programmed cell death protein-1 (PD-1) inhibits the activation and function of T cells. When PD-1 on activated T cells binds to programmed cell death ligand 1 (PD-L1) or programmed cell death ligand 2 (PD-L2) on tumor cells, T cells become inactivated, allowing tumor cells to evade the host immune response. Anti-PD-1 or PD-L1 antibodies can inhibit suppressive effects of tumor cells and enhance antitumor activities. **b** T-cell activation requires both TCR signaling and co-stimulatory signaling (CD28). When T cells get activated, cytotoxic T-lymphocyte-associated protein 4 (CTLA-4) is upregulated and displaced by CD28 binding to B7 due to higher binding affinity. The binding of B7 proteins to CTLA-4 inhibits T-cell activation

Immune checkpoints are regulators of the immune system and play an important role in preventing self-attacks by the immune system^24^. Malignant cells usually express unique antigens, which allow our immune system to differentiate them from our normal cells and subsequently remove them. However, many tumor cells have evolved a mechanism to escape from the host immune system, by expressing cell surface molecules, which interact with immune checkpoint receptors on T cells causing the T cells to erroneously classify the tumor cells as healthy normal cells. Hence, inhibiting these checkpoint receptors (on either the host T cells or the tumor cells) can potentially be an effective means for allowing the host T cells to again properly classify tumor cells. Well-known immune checkpoint inhibitors include those that target programmed cell death protein 1 (PD-1, also known as CD279) or cytotoxic T-lymphocyte-associated protein 4 (CTLA-4, also known as CD152) (Fig. 1a, b)^25, 26^. Already, a significant number of clinical studies have been conducted using this strategy for cancer therapy and have demonstrated efficacy of immune checkpoint blockades in a variety of solid tumors and hematologic malignancies^27^.

### Overview of PDX models

In vitro methods for testing anticancer drugs can employ monolayer cell cultures or organoid cultures and are especially beneficial in genetic modification and high-throughput screening assays. However, these methods have some limitations such as the selective proliferation of clonal cells^28^. PDX models are thought to overcome some of these limitations and seem to preserve key characteristics of the patient’s tumors, including histological features, genomic signatures, and the genetic heterogeneity of cells in a tumor mass^29^. Therefore, they seem to recapitulate many aspects of the original patient tumor and can potentially serve as a more accurate predictive platform for therapeutic outcomes^30^.

### Generation of the PDX models

**Fig. 2:**
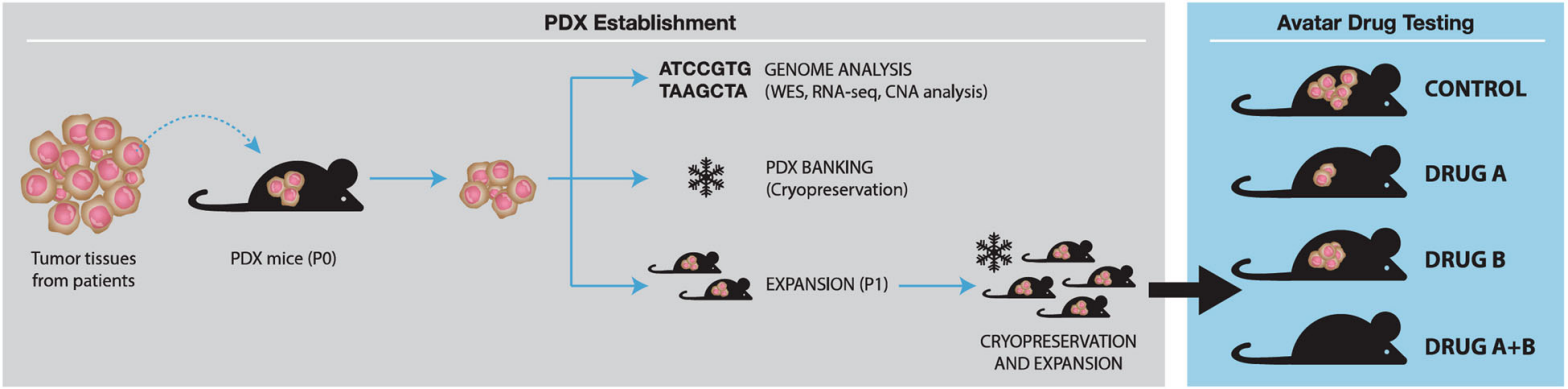
Generation of PDX models for chemotherapy. Tumor tissues from cancer patients are implanted into immunodeficient mice (P0) subcutaneously or orthotopically. After the growth of tumor tissues in P0 mice, these tissues are used for genomic analysis such as whole exome sequencing (WES), RNA sequencing (RNA-seq) and copy number alteration (CNA) analysis and can be preserved or reimplanted into new mice for tumor cell passaging. After more expanding of these tumor xenografts, in vivo drug response testing can be performed in these models

PDX models are generated by the implantation of fresh human tumor tissues into immunodeficient mice, to reduce rejection of the tumor cells by the mouse. Tumor tissues, no larger than 2 mm^3^, are implanted into immunodeficient mice either subcutaneously or orthotopically (i.e., at the same tissue-of-origin site). One or two fragments are generally implanted into each mouse. The tumor masses are then grown to ~1000 mm^3^ in size, at which point, the tumors can be cryopreserved, characterized, or dissected again for reimplantation and propagation in additional mice (Fig. 2). For hematological cancers, PBMCs or bone marrow samples from patients with leukemia are engrafted either in the blood stream or into the bone marrow^31, 32^. The continued propagation of the human tumors in the immunodeficient mice make PDX models a renewable resource for cancer studies.

There are a variety of immunodeficient mice that can be used for PDX models: severely compromised immune-deficient (SCID) mice, athymic nude mice, non-obese diabetic (NOD)–SCID mice, and recombination-activating gene 2 (Rag2)-knockout mice^33^. More recently, NOD.Cg-*Prkdc*^scid^ *Il2rg*^tm1Wjl/SzJ^(NSG) mice have become the mouse strain of choice for such PDX studies because this mouse has no IL-2 receptor gamma, which is an important component of the surface receptor of immune cells that transduce signals from six kinds of interleukins. Since the signaling pathway of IL-2 receptor gamma is needed for the differentiation and function of many hematopoietic cells, the absence of this receptor leads to a dysfunction in innate immunity, including natural killer (NK) cells. This makes the NSG mouse a very effective model for the engraftment of primary tumor tissues or cells^34, 35^.

## Applications of PDX models in cancer research

### PDX models for exploring drug efficacy and the mechanism of resistance

PDX models have already been used in a diverse range of preclinical cancer research projects. For example, a recent study using PDXs demonstrated the inhibition of the nuclear exporter for antitumor efficacy in a triple-negative breast cancer^36^. In another study, lung cancer PDX models revealed the antitumor activity of kinetochore-associated protein 2 siRNA^37^. Similarly, another study demonstrated the antitumor effects of multikinase inhibitors in PDX models of hepatocellular carcinoma^38^.

One problem that continues to be explored is how well the drug efficacy data using these PDX models correlate with actual clinical outcomes^39^. Several studies have been published that seem to suggest that in some cases, there can be good correlations between the two. For example, one study showed a high correlation between PDX models and clinical trials for over 3300 drug response datasets^40^. In another study, PDX models of colorectal cancers treated with an epidermal growth factor receptor inhibitor, cetuximab, showed comparable response rates to those of the patients in whom the tumor orginated^41^. Finally, responses to sirolimus, sunitinib, and dovitinib, but not erlotinib, were largely correlated between PDX models and corresponding clinical outcome results for renal cell cancers^42^.

Having a good correlation between PDX models and clinical trials provides a chance to find novel biomarkers for drug reactivity. For example, in a melanoma PDX model introducing vemurafenib resistance, the resistant tumors showed dependency on BRAF signaling due to the elevated BRAF (V600E) expression^43^. These data suggest the possibility that elevated BRAF expression could be used as a biomarker for vemurafenib resistance^43^. Another study found a molecular mechanism for gemcitabine resistance through the use of PDX models for pancreatic cancer^44^.

## PDX models for co-clinical trials

Co-clinical trials are preclinical research studies that can be conducted in parallel with human patient treatments in the clinical setting. In this application, PDX models are generated from cancer tissues of patients in clinical trials, and the PDXs are treated with the same and possibly additional therapies to follow clinical responses^45^. The responses to new agents, mechanisms underlying the responses to treatment, and explorations of prognostic biomarkers can be studied by using established PDXs from patients being treated. Using such co-clinical trials, strategies for new combinations can also be suggested. For example, a phase II co-clinical trial of arsenic trioxide in relapsed small cell lung cancer revealed that PDX modeling reliably reproduced clinical outcomes^46^. Another recent study revealed that the response to dovitinib in lung squamous cell carcinomas could be predicted by signatures of FGFR gene expression^47^. Such co-clinical trials provide a chance to evaluate the efficacies of several drugs, in isolation or combination, in a cost-effective and cost-efficient manner.

### Limitations of PDX Models

Although PDX models are excellent in vivo platforms for precision medicine, they have several limitations that should be noted. First, the development of a PDX model from a patient can take as long as 6 months (or longer) to establish. In addition, certain tumor types, such as prostate cancers, are difficult to establish as PDX models, presumably because of the need for additional, unknown “factors” needed for proper tumor growth. Finally, those tumors that have genetic heterogeneity cannot always be recapitulated in serial passages if the genetic heterogeneity is not all represented in the dissected tumor that is passaged.

### Humanized mice with PDXs: a groundbreaking research platform for cancer immunology

As outlined above, PDX models can be a useful resource for preclinical trials; however, these are limited to chemotherapeutic drugs. For immunotherapeutic options, an intact immune system is required. The use of mice with a murine immune system is limiting, as it does not accurately recapitulate the human immune system. Therefore, creating a mouse with a human immune system (a “humanized mouse”) is a better vehicle for immunotherapeutic efficacy testing.

### Creating humanized mice: an in vivo model that uses human immune systems

**Fig. 3:**
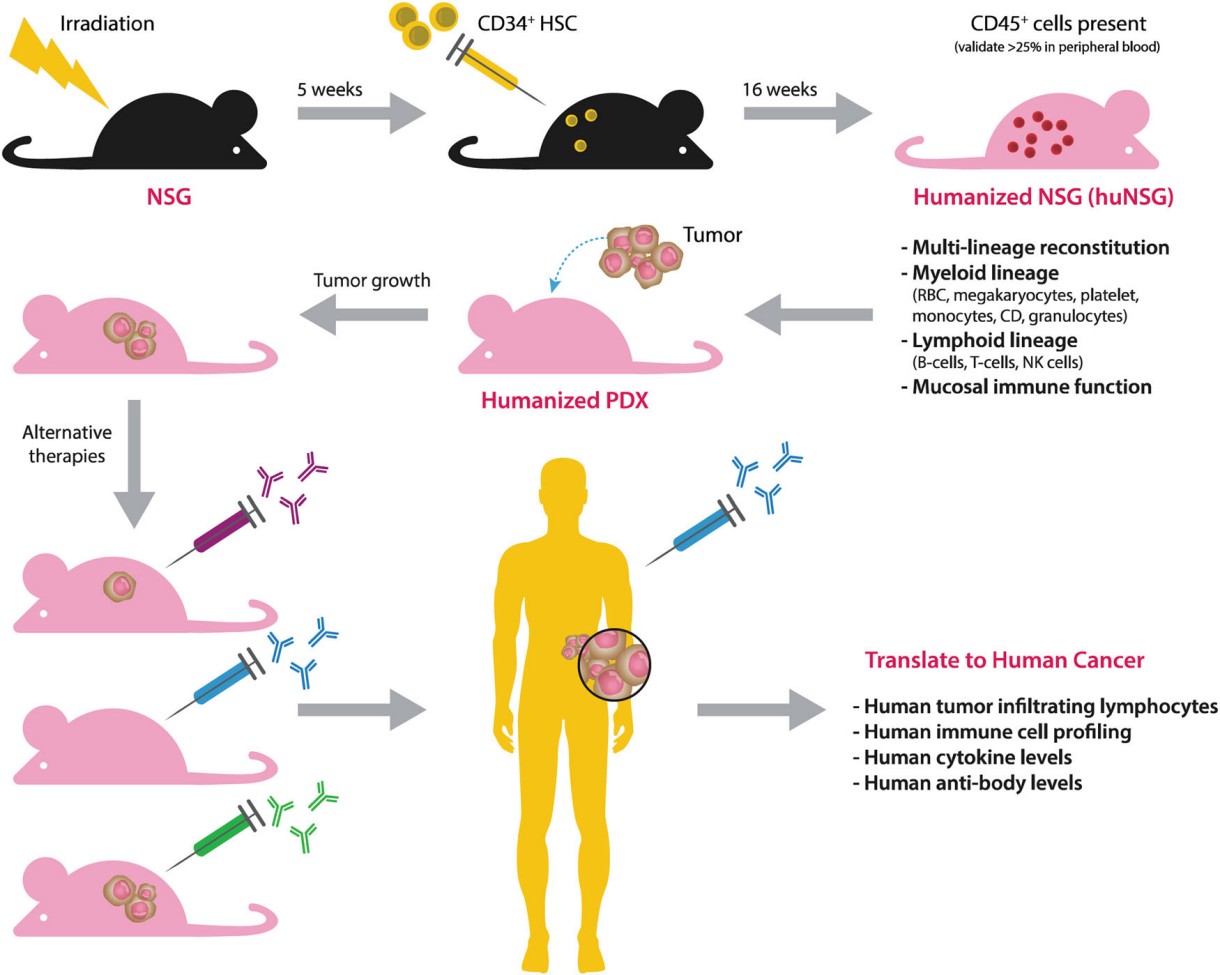
Generation of humanized mice for immunotherapy models. The NSG mice are irradiated with whole body gamma irradiation. Human CD34^+^ hematopoietic stem cells (HSCs) are intravascularly injected into NSG mice at 5 weeks. Humanization for the engraftment of human HSCs is monitored by flow cytometry of use for determining the percentage of differentiated human CD45^+^ cells in the peripheral blood of the mice. Patient tumor tissues are then engrafted into ‘humanized’ mice and used for studying the efficacy of variety therapies. These humanized PDX models can also permit the study of human immune responses, including the analysis of TILs, cytokines, and antibodies

The ultimate goal of humanization is to generate mice with a fully competent human immune system, capable of mounting anticancer immune responses for specific immunotherapeutic interventions (Fig. 3). In its most basic form, the NSG mouse can be engrafted with various types of human leukocytes and purified human CD34^+^ hematopoietic stem cells (HSCs), obtained from bone marrow, umbilical cord blood, fetal livers or thymus tissues^48^.

Humanized PDX models are generated by implanting fresh human tumor fragments into these humanized mice. Initially, NSG mice that are 5 to 12 weeks old are first irradiated with 50 to 250 cGy whole body gamma irradiation to enhance engraftment. Then, 3 × 10^4^–1 × 10^5^ human CD34^+^ HSCs are intravascularly injected into each irradiated NSG mouse. At approximately 10–12 weeks of age, engraftment of the human HSCs can be confirmed by assessing for differentiated human CD45^+^ cells (leukocyte common antigen) in the peripheral blood of the mice using flow cytometry. Human CD45^+^ cells can be detected as early as 4 weeks after the engraftment of HSCs^49^. Successful engraftment of a human immune system can be considered when the mice have more than 25% human CD45^+^ cells in their peripheral blood. The process for generating humanized mice is summarized in Fig. 3. Specific PDXs can then be inserted into the humanized mice, and an immunotherapeutic agent is subsequently applied for testing. Afterward, the host immune response to an immunotherapeutic agent can be analyzed using different methods (Fig. 3).

To produce humanized mice effectively, several immunodeficient mouse strains have been developed. For example, the NOD.Cg-*Prkdc*^scid^ *Il2rg*^tm1Wjl^ Tg(CMV IL-3, CSF2, KITLG)1Eav/MloySzJ (also known as NSG-SGM3) mice expresses human IL-3, Granulocyte-macrophage colony-stimulating factor (GM-CSF) and stem cell factor allows for the stable engraftment of human HSCs for humanization^50^. In addition, the NOD,B6.SCID *Il2r*γ^−^^/−^ *Kit*^W41/W41^ (NBSGW) mice, which carry mutations in c-Kit, support the transplantation of HSCs without irradiation^51, 52^. The human *SIRPA* and *IL15* knockin (SRG-15) mice also showed an increased development of intraepithelial lymphocytes, innate lymphoid cell subsets and NK cells^53^.

Ideally, the humanized mice being used would have the same immune system from which the PDX was derived. However, it is very difficult to obtain CD34^+^ cells from the cancer patient, and therefore, an allogenic immune approach is usually used.

## Application of humanized mice with PDXs for cancer research

### The tumor microenvironment

The relationship between the tumor and the surrounding stromal and immune cells is highly complex and an area of exciting research^54^. Indeed, the interplay between the rapidly dividing cancer cells and the surrounding stromal tissue is thought to be one critical factor in treatment efficacy^55^. Cancer cells can interact with the stromal microenvironment in several ways. (1) As cancer cells divide, they can recruit cells that contribute to the infrastructure of the growing tumor, including fibroblasts, endothelial cells, and circulating immune cells^56^. Chemokines released from cancer cells, stromal cells and leukocytes can regulate angiogenesis. Cancer cells also produce cytokines (protein messengers that tell other cells when and where to launch an immune response) to control nearby immune cells and help them escape immune surveillance. Using humanized mice, Morton and colleagues reported that human immune cells maintained the microenvironment of engrafted cancer PDXs^57^. The human immune cells, which infiltrated the engrafted tumors, induce lymphangiogenesis and sustain the original gene expression profile of the PDX^57^. This study indicates that interactions between stromal immune cells and tumor cells are indeed important in maintaining the integrity of the original tumor.

### Humanized mouse model for cancer immunotherapy

Numerous recent studies have now demonstrated the advantages and advancements of cancer immunotherapy using humanized mice. For example, in a recent study, a renal cell carcinoma (RCC) mouse model was generated orthotopically with PBMCs to evaluate the antibody efficacy targeting the carbonic anhydrate IX protein in RCC. This study demonstrated that the antibody inhibited cancer growth by priming T-cell activity^58^. In October 2017, Akiyama and colleagues demonstrated that the STAT3 inhibitor, STX-0119, had an antitumor activity, whereby lymphocytes accumulated within the engrafted tumor tissues of the humanized mice^59^. Similarly, another study demonstrated that a PD-1 inhibitor could effectively restrain osteosarcoma pulmonary metastasis^60^. Finally, Hu Z et al., collected T cells from humanized mice, and found these T cells to have cytotoxic activity to melanoma cells in vitro in an antigen-specific manner. The transfer of these T cells to a melanoma-bearing PDX model substantially extended the animal’s survival^61^. This study demonstrates that humanized mice are a useful resource to study the functions of antigen-specific T cells for cancer immunotherapy.

Humanized mouse models can also be used to study the complicated relationship between tumor development, oncolytic viruses, and the human immune system. Tsoneva and colleagues placed lung carcinomas into humanized mice to determine the interaction of the oncolytic vaccinia virus (VACV) with the host immune system and how it affects tumor growth^62^. They validated the efficacy of combination therapy using CTLA4-blocking antibody and VACV, which was only possible with the humanized mouse model^62^.

Humanized mouse models have also been used to study the efficacy of ACT treatments. For example, to study ACT and the immune checkpoint blockade, Jespersen and colleagues inserted melanoma tumor cells and T cells from the same patient into humanized mice and showed tumor inhibition^63^. Interestingly, T cells from ACT non-responders failed to inhibit tumor growth in the same model^63^. It would be important to explore more studies for many other types of well-characterized human cancers.

### Human microbiota-associated mice

The microbiota comprises commensal and other microorganisms that inhabit the epithelial barriers of the host body^64^. The microbiota regulates many physiological functions in the host body, including metabolism, hematopoiesis and immunity^65^. The homeostatic interaction with these microorganisms is particularly important for the development of the host immune system^65–67^. However, commensal microorganisms exist in a balance that, if significantly altered, enters dysbiosis, which can be associated with the etiology of many types of cancers^68–72^. Indeed, recent studies have shown some evidence that the microbiota, especially within the gut, can impact the efficacy of certain cancer therapies, including chemotherapy, radiotherapy and immunotherapy^73^. Considering the importance of the microbiota, further studies of the effects of the microbiota on biological responses would be useful.

Because germ-free mice have no microbes, researchers have tried to control the microbiota through direct inoculation with a specific microorganism or combinations of them^64^. Therefore, researchers have developed human microbiota-associated mice that are established from germ-free mice by fecal microbiota transplantation^74, 75^. This method has been used to study the interaction between the host and microorganism as well as the effects of microbes on human health and diseases, including cancers^64^.

## Future directions

In this review, we have shown many advantages for the use of humanized mice in studying cancers. In particular, there is an ongoing need to generate more comprehensive and functional immune systems within these humanized mice and identify new practical approaches that would enable autologous experiments that engraft diseased tissues and immune cells from the same individual for a more accurate understanding of disease progression and personalized treatment efficacy. Despite the noted limitations, the use of PDX models in humanized mice has already provided many insights into the behaviors of diverse cancers within their native tumor microenvironments, under the effects of human immune cells. As humanized PDX models continue to improve and truly recapitulate the human biological system, these models will provide an unprecedented research platform in cancer immunology and personalized medicine.
